# In silico conversion of ssRNA aptamers to ssDNA: molecular dynamics assessment of structural stability and conformational preservation

**DOI:** 10.1007/s10822-026-00775-9

**Published:** 2026-02-23

**Authors:** Sabrina Lorenti, Nathalia Oliveira Alqualo, Danilo Caixeta Moreira, Nilson Nicolau Junior, Vivian Alonso Goulart

**Affiliations:** 1 Laboratory of Nanobiotechnology Prof. Dr. Luiz Ricardo Goulart Filho, Institute of Biotechnology, Federal University of Uberlândia, Umuarama Campus, Avenida Pará, 1720, 38.400-902, Uberlândia, Minas Gerais Brazil; 2 Laboratory of Molecular Modeling, Institute of Biotechnology, Federal University of Uberlândia, Umuarama Campus, Avenida Pará, 1720, 38.400-902, Uberlândia, Minas Gerais Brazil

**Keywords:** Prostate cancer, ssRNA and ssDNA aptamers, Molecular dynamics simulation, Structural stability, In silico conversion, Computational biotechnology

## Abstract

The application of single-stranded RNA (ssRNA) aptamers may be limited by their chemical instability and susceptibility to enzymatic degradation, despite their high structural specificity. Thus, this study presents a computational workflow for the rational conversion of ssRNA aptamers (A6 and A11), previously selected for prostate cancer cells, into structurally preserved single-stranded DNA (ssDNA) analogues, termed LN-A6 and LN-A11. The workflow integrates three-dimensional structural modeling, targeted chemical modifications, and molecular dynamics simulations conducted for 200 ns at 300 K and 310 K, aiming to assess conformational preservation under different thermal conditions. Structural comparisons between ssRNA and ssDNA were performed using widely adopted molecular dynamics descriptors, including root-mean-square deviation and the number of intramolecular hydrogen bonds throughout the simulation trajectories. The results indicate that the ssDNA variants retained key structural features of their ssRNA precursors, exhibiting consistent conformational behavior at both analyzed temperatures. Although the ssRNA aptamers displayed more conformationally restricted architectures, the ssDNA analogues preserved sufficient structural integrity to support the feasibility of the conversion from a conformational standpoint. Overall, this study describes a reproducible computational workflow for evaluating structural preservation during the conversion of ssRNA to ssDNA aptamers, providing a methodological foundation for future experimental investigations.

## Introduction

The growing demand for highly specific molecular tools in prostate cancer has positioned aptamers as valuable synthetic ligands for both diagnostic and therapeutic applications. Recent evidence indicates that aptamer-based platforms can be successfully applied for biomarker detection [1]. These oligonucleotides exhibit high affinity and specificity for their molecular targets, along with low immunogenicity and chemical versatility, features that make them particularly attractive for the selective recognition of cellular biomarkers, with direct implications for disease diagnosis, monitoring, and management [2, 3]. Among the different classes, single-stranded RNA (ssRNA) aptamers have been extensively explored due to the robustness of selection methodologies, such as SELEX, and the availability of well-established computational tools for structural prediction. However, limitations related to chemical stability and susceptibility to ribonuclease-mediated degradation restrict their translational applicability. Therefore, the conversion of ssRNA into single-stranded DNA (ssDNA) has emerged as a promising strategy, as ssDNA molecules exhibit greater stability, practical viability, and compatibility with clinical and technological applications [4].

Despite these advantages, the rational conversion of ssRNA aptamers into ssDNA counterparts represents a significant structural challenge. Unlike ssRNA molecules, for which relatively well-established computational pipelines exist for three-dimensional (3D) structure prediction, there is currently no single, direct computational tool capable of reliably generating 3D models of ssDNA aptamers. The structural modeling of these molecules often relies on indirect approaches, in which secondary structure prediction – a step highly sensitive to nucleotide composition and to the computational algorithm employed – plays a decisive role in determining the accuracy of subsequent three-dimensional models [5, 6].

A previous study demonstrated that even for single-stranded DNA structures, such as hairpins, the generation of reliable three-dimensional models requires multistep strategies that combine the initial construction of RNA-inspired architectures with refinement through molecular dynamics simulations. These findings highlight that the absence of a dedicated computational framework for ssDNA necessitates indirect approaches, in which RNA-derived models are used as structural references to enable three-dimensional modeling [2].

Reinforcing this challenge, Bachu et al. [7] conducted a systematic analysis of the limitations associated with three-dimensional modeling of ssDNA aptamers, revealing that reliable models cannot be obtained through direct methods but instead depend critically on secondary structure prediction. This step was shown to be particularly sensitive to nucleotide composition, especially guanine content and GC percentage, as well as to the computational algorithm employed, further underscoring the need for indirect strategies based on RNA-derived structures.

Thus, this study aims to evaluate the structural conversion of two ssRNA aptamers, A6 and A11, into their ssDNA counterparts through three-dimensional modeling and molecular dynamics simulations. These aptamers, previously selected using the 3D Cell-SELEX methodology against the PC-3 prostate cancer cell line [8], were used here as model systems to investigate conformational preservation following RNA-to-DNA conversion.

## Methodology

### In silico modification of ssRNA to ssDNA aptamers

The ssRNA sequences of aptamers A6 and A11, originally reported by Souza et al. [8], were subjected to nucleotide substitution, where ribonucleotides were replaced with deoxyribonucleotides to enhance molecular stability.

Initially, the secondary structures of the ssRNA oligonucleotides were predicted using the Mfold algorithm. Based on the resulting 2D conformations, 3D structural models were generated using the RNAComposer platform.

Subsequently, the ssRNA 3D structures were modified into ssDNA structures following the protocol described by Jeddi and Saiz [2]. This process involved: (I) removing the 2’-OH hydroxyl group from the ribose sugar to simulate the deoxyribose structure; (II) replacing the hydrogen atom at the 5’ position of uracil with a methyl group (–CH₃), thus converting it into thymine, using the Molefacture plugin in VMD; and (III) manually renaming the modified uracil residues to thymine in the PDB file. These steps were sequentially applied to preserve the original ssRNA conformation and to generate structurally accurate ssDNA models suitable for molecular dynamics simulations.

**Fig. 1:**
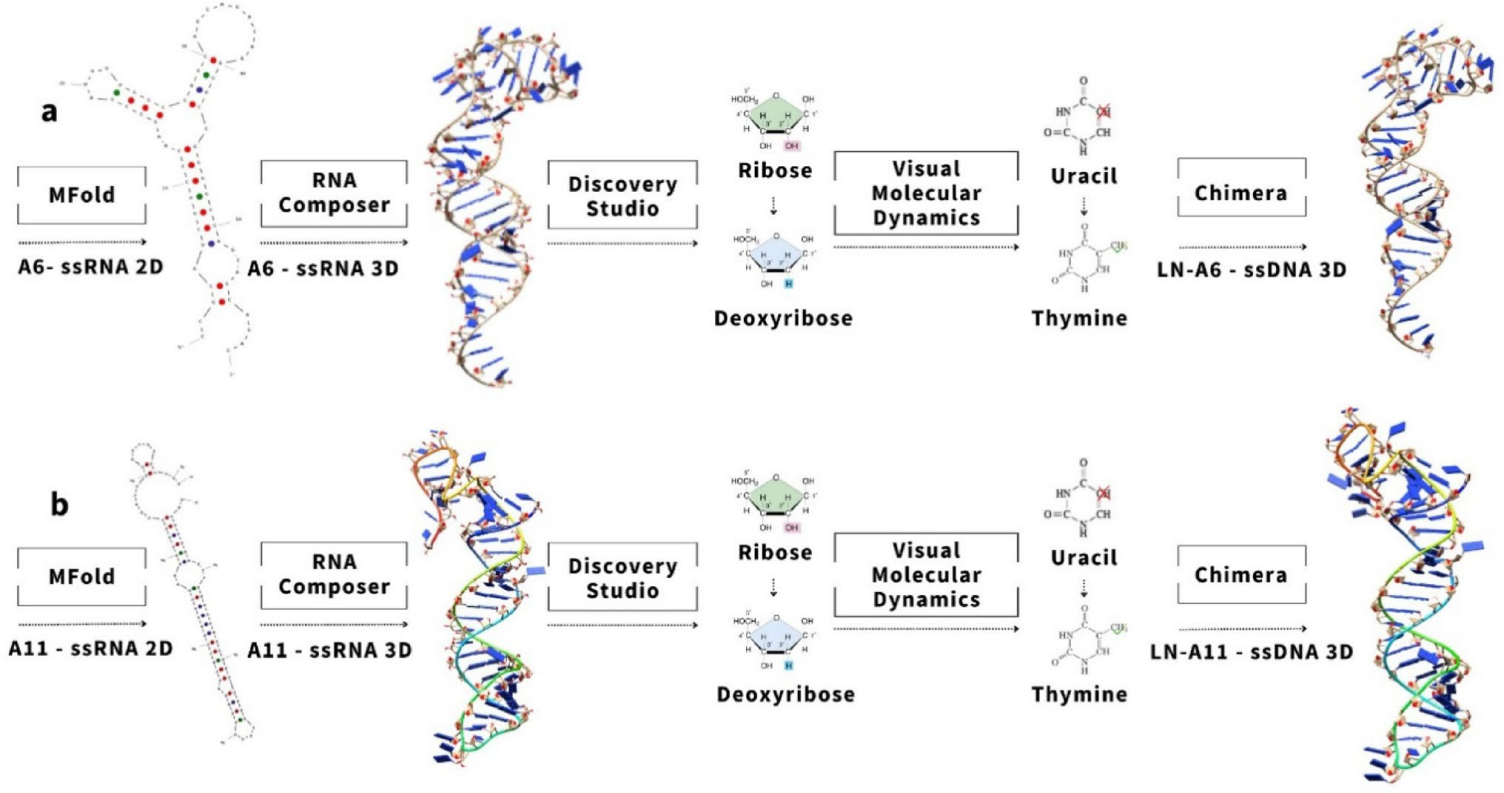
Modeling of ssRNA aptamers into ssDNA. **a** A6 ssRNA modified into LN-A6 ssDNA; **b** A11 ssRNA modified into LN-A11 ssDNA

Lastly, the 3D structures of the modified ssDNA aptamers were successfully visualized and analyzed using UCSF Chimera [4] (Fig. 1).

### Molecular dynamics and comparison of SsRNA and SsDNA aptamers

**Fig. 2:**
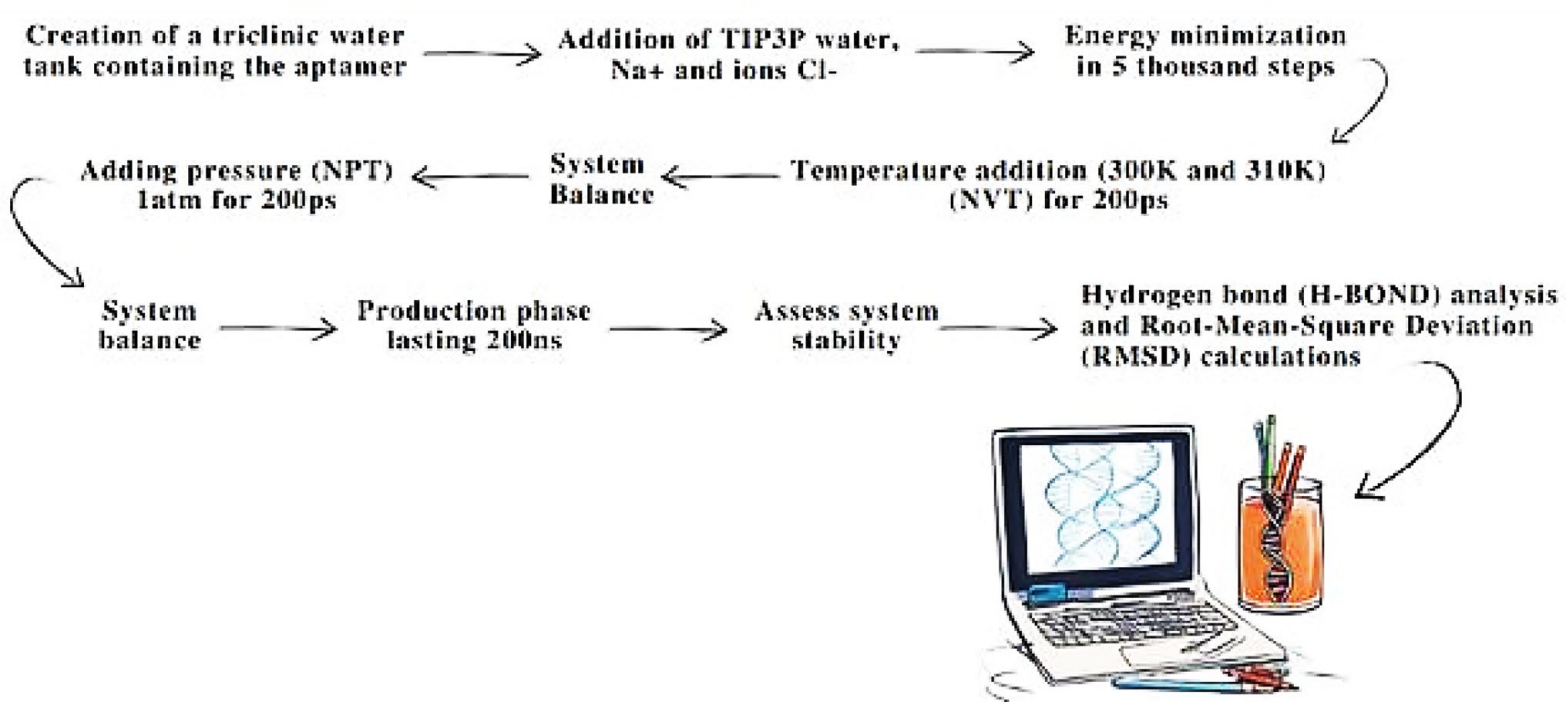
Steps of molecular dynamics and post-dynamics analyses using ssDNA aptamers LN-A6 and LN-A11

After modifying aptamers A6 and A11 from ssRNA into their ssDNA counterparts, designated LN-A6 and LN-A11 (Fig. 1a and b), we carried out molecular dynamics (MD) simulation studies for both nucleic acid structures using the GROMACS software package [3]. The MD simulations for the ssDNA molecules (Fig. 2) were performed using the CHARMM27 force field combined with the TIP3P explicit water model [9]. The simulation box was configured with a triclinic geometry. Water molecules and counterions (Na⁺ and Cl⁻) were added to neutralize the system, and energy minimization was carried out using the steepest descent algorithm.

System equilibration was conducted in two consecutive steps: (I) under an NVT ensemble (constant volume and temperature) and (II) under an NPT ensemble (constant pressure and temperature), each performed for 100 ps. These equilibration steps were followed by a 200 ns production run under NPT conditions. The Particle Mesh Ewald (PME) method was used to calculate long-range electrostatic interactions during the simulations [10].

**Fig. 3:**
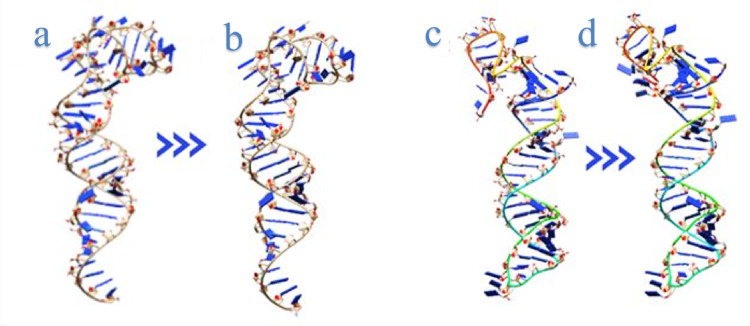
ssRNA and ssDNA aptamers: **a** RNA A6; **b** ssDNA LN-A6; **c** RNA A11; **d** ssDNA LN-A11

Each ssDNA system was simulated in duplicate, with temperature as the sole experimental variable. Simulations were performed at 300 K and 310 K, using a modified Berendsen thermostat for temperature coupling [10]. These temperatures were selected to evaluate the structural robustness of the aptamers under different thermal conditions, allowing the investigation of how moderate variations in kinetic energy influence molecular conformational stability. Pressure was maintained at 1 atm using the Parrinello–Rahman barostat [11], in order to reproduce standard isothermal–isobaric conditions widely employed in biomolecular simulations. Short-range electrostatic and van der Waals interaction cutoff distances were set to 1.2 nm, in accordance with established parameters to ensure an appropriate balance between computational efficiency and accurate description of intermolecular interactions (Fig. 3).

MD trajectories were analyzed using integrated tools within the GROMACS package. Structural stability was assessed through root-mean-square deviation (RMSD) analysis to quantify conformational changes in the aptamers over time. Additionally, a hydrogen bond (H-bond) analysis was performed to determine the number of internal hydrogen bonds formed within each aptamer during the simulations.

## Results and discussion

### SsRNA to SsDNA aptamers show similar 3D structures

**Fig. 4:**
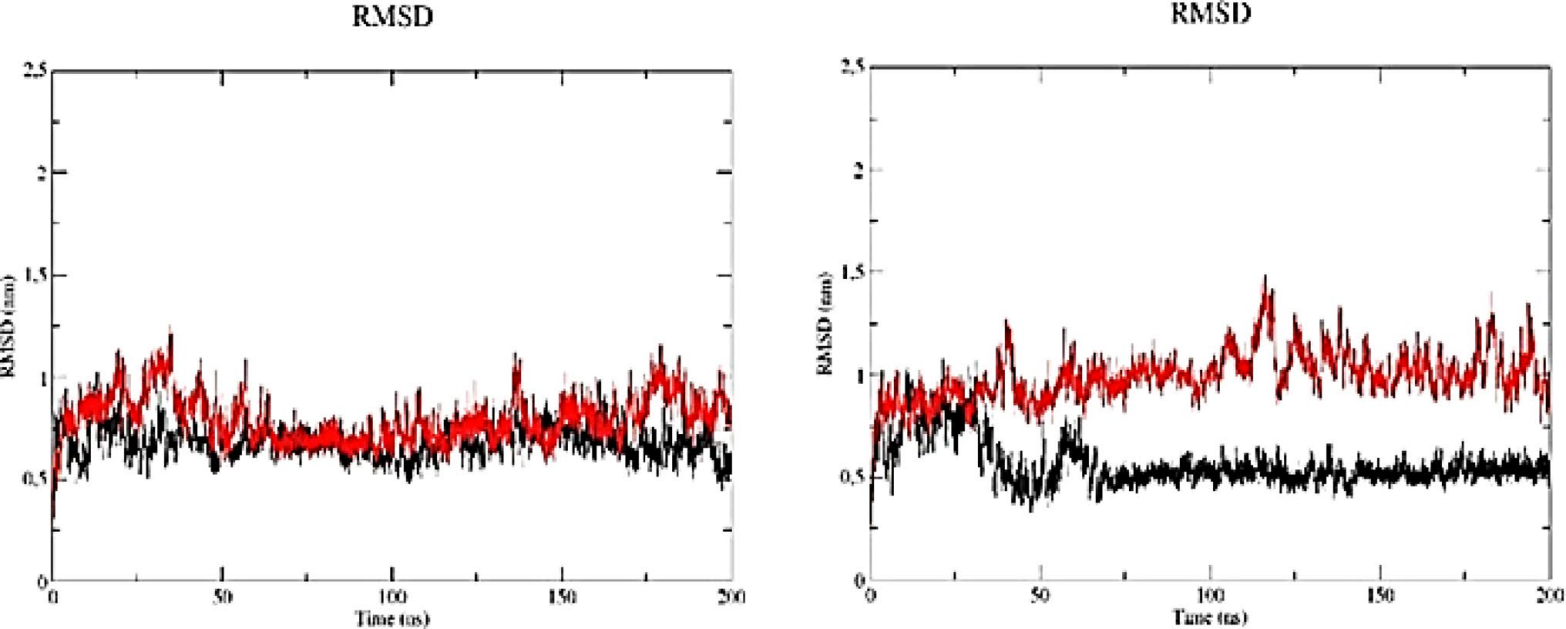
RMSD profiles of A6 and LN-A6 during molecular dynamics simulations at **a** 300 K (26 °C) and **b** 310 K (36 °C). A6 is shown in black and LN-A6 in red

The redesigned oligonucleotide structures remained structurally similar to their original ssRNA counterparts (Fig. 4), potentially indicating that the ssDNA aptamers preserved their binding affinity toward the PC-3 cell line. This step was specifically aimed at maintaining the conformational architecture as close as possible to that of the native ssRNA aptamers, in order to preserve structural features relevant to molecular recognition.

Besides modeling the ssRNA structures, we generated the corresponding ssDNA structures for each aptamer by substituting uracil (U) bases with thymine (T). The original RNA sequences – RNA A6: 5′ CCG CAU CGU CCC AAG CCG AUU UUG GCG AGC AGC AGA CAG GUU CCG GGG CGA GCA GCA GAC 3′ and RNA A11: 5′ CGA UGC GGA AUA GGG GCC AGG CGUAUC UGA GCU CCU AUU CUC UUU GUC CCG UCU GCU GCU 3′ – were refined to the following DNA sequences with uracil replaced by thymine:

LN-A6 – DNA: 5′ CCG CAT CGT CCC AAG CCG ATT TTG GCG AGC AGC AGA CAG GTT CCG GGG CGA GCA GCA GAC 3′.

LN-A11 – DNA: 5′ CGA TGC GGA ATA GGG GCC AGG CGT ATC TGA GCT ATT CTC TTT GTC CCG TCT GCT GCT 3′.

Structural modeling of ssDNA aptamers represents an essential step for conformational analysis prior to experimental validation, as it enables the generation of physically plausible three-dimensional structures. Methodological approaches similar to those employed in the present study have been developed to establish and validate computational strategies for generating realistic three-dimensional models of ssDNA hairpins, which constitute key structural motifs in aptamers used in biosensor applications [2].

### Molecular dynamics analysis

MD simulation results allowed the analysis of the RMSD of the studied molecules. This metric quantifies variations in atomic coordinates throughout the simulation and serves as an estimate of structural changes over time. Lower RMSD values indicate minimal conformational fluctuations and, consequently, greater structural stability.

The observation of an RMSD plateau for the A6 aptamer at 310 K after approximately 75 ns (Fig. 4; Table 1) suggests that the trajectory reached a relatively stationary conformational regime with respect to the reference structure, indicating stabilization of structural deviation over time.

**Table 1:** Means and standard deviations of H-bond and RMSD analyses for ssRNA and ssDNA aptamers. Analyses were conducted at 300 K (black) and 310 K (red)

HB number			RMSD	
	Average	SD	Average	SD
ssRNA				
A6 (300 K)	79.85	5.02	0.56	0.11
A6 (310 K)	81.09	5.13	0.69	0.08
A11 (300 K)	94.31	5.59	1.27	0.15
A11 (310 K)	95.85	6.00	0.69	0.08
ssDNA				
LN-A6 (300 K)	59.15	3.99	0.99	0.12
LN-A6 (310 K)	60.72	4.45	0.79	0.12
LN-A11 (300 K)	72.11	3.87	1.69	0.20
LN-A11 (310 K)	66.07	3.95	1.30	0.17

SD = standard deviation

This interpretation is widely adopted in molecular dynamics studies, where RMSD plateauing is commonly used as a practical indicator of structural stabilization over time [12, 13]. The conformational stabilization observed, particularly for the A6 aptamer, indicates that the molecule attained a relatively stationary conformational regime during the simulation, characterized by lower-amplitude RMSD fluctuations compared with its ssDNA counterpart [14]. This behavior reflects the maintenance of a coherent three-dimensional architecture relative to the reference structure, with no evidence of structural drift over time. From a structural perspective, the preservation of specific conformational motifs, such as loops, stems, and base-stacking regions, is a relevant aspect, as aptamer-mediated molecular recognition is largely dependent on their three-dimensional spatial organization [15, 16]. In this sense, the maintenance of a stable conformation throughout the simulation indicates conservation of structural elements potentially associated with target recognition, without implying direct functional inference.

Furthermore, the relatively higher stability observed for A6 compared with LN-A6 supports the hypothesis that aptamers initially selected in the ssRNA form tend to exhibit more complex conformational architectures, which may be partially preserved following conversion to ssDNA, provided that structural integrity is maintained during the conversion process [17]. In contrast, LN-A6 exhibited more pronounced RMSD fluctuations throughout the simulation, indicating greater conformational flexibility under the same thermal conditions.

**Fig. 5:**
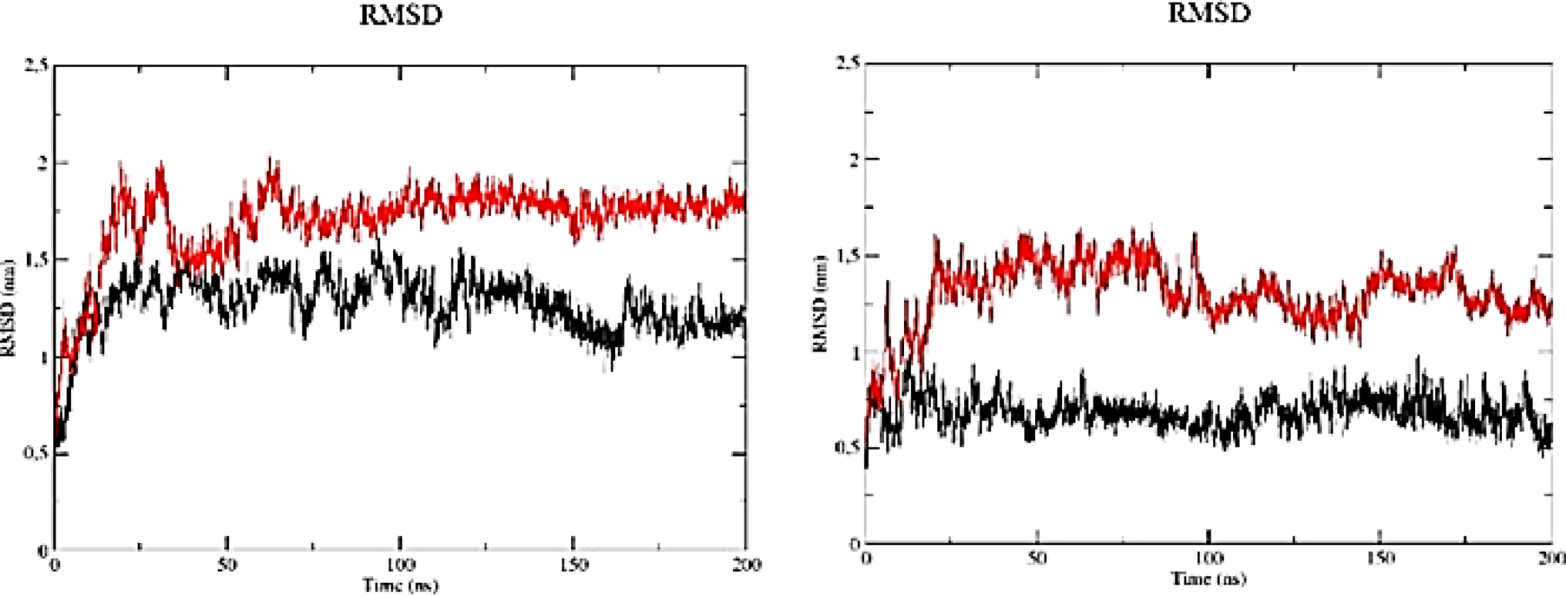
RMSD profiles of A11 and LN-A11 during molecular dynamics simulations at **a** 300 K (26 °C) and **b** 310 K (36 °C). A11 is shown in black and LN-A11 in red

Analysis of the RMSD profiles of the A11 and LN-A11 aptamers at 300 K revealed that A11 exhibited lower mean RMSD values and standard deviations compared with LN-A11 (Fig. 5; Table 1). Although LN-A11 displayed a higher average RMSD, its fluctuations became less pronounced after approximately 100 ns, suggesting stabilization within a relatively stationary conformational regime around a higher plateau. In contrast, A11 maintained lower RMSD values but exhibited a slight tendency toward variation along the trajectory, without a plateau as well defined as that observed for LN-A11.

**Fig. 6:**
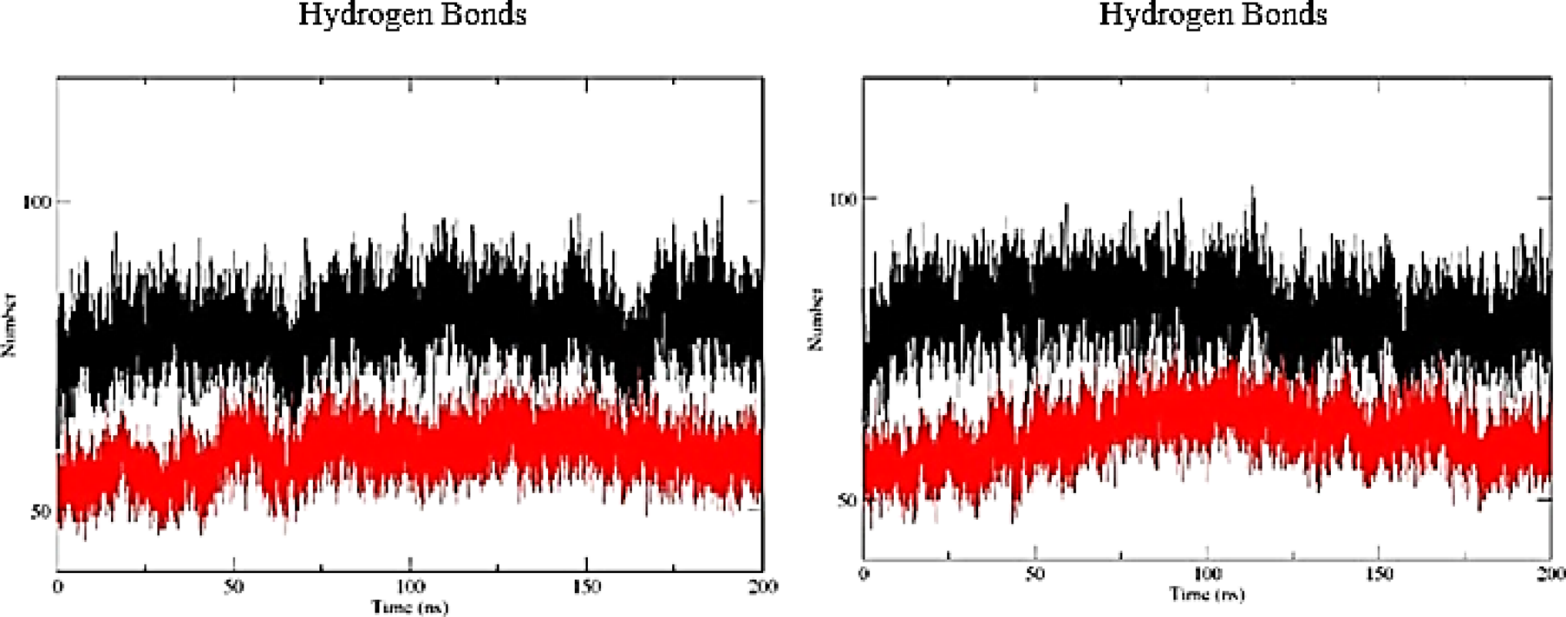
Time evolution of intramolecular hydrogen bonds for A6 and LN-A6 at **a** 300 K (26 °C) and **b** 310 K (36 °C). A6 is shown in black and LN-A6 in red

At 310 K, both aptamers exhibited RMSD profiles without evidence of pronounced structural drift, indicating relatively stationary conformational behavior under this thermal condition. In this context, the A11 aptamer consistently maintained lower mean RMSD values than those observed for LN-A11 (Fig. 6; Table 1), reflecting reduced structural deviation from the reference structure throughout the simulation.

In structural analyses, the presence and persistence of intramolecular hydrogen bonds may contribute to conformational stability by supporting maintenance of the molecule’s three-dimensional organization. The A6 aptamer exhibited a higher average number of intramolecular hydrogen bonds compared with LN-A6, with mean values of 79.85 and 81.09 at 300 K and 310 K, respectively (Table 1), indicating a more extensive interaction network and a structurally more constrained architecture throughout the simulation. In contrast, LN-A6 showed greater temporal variability in the number of hydrogen bonds, particularly at 310 K (Fig. 6), reflecting increased conformational flexibility, without implying direct functional inference.

**Fig. 7:**
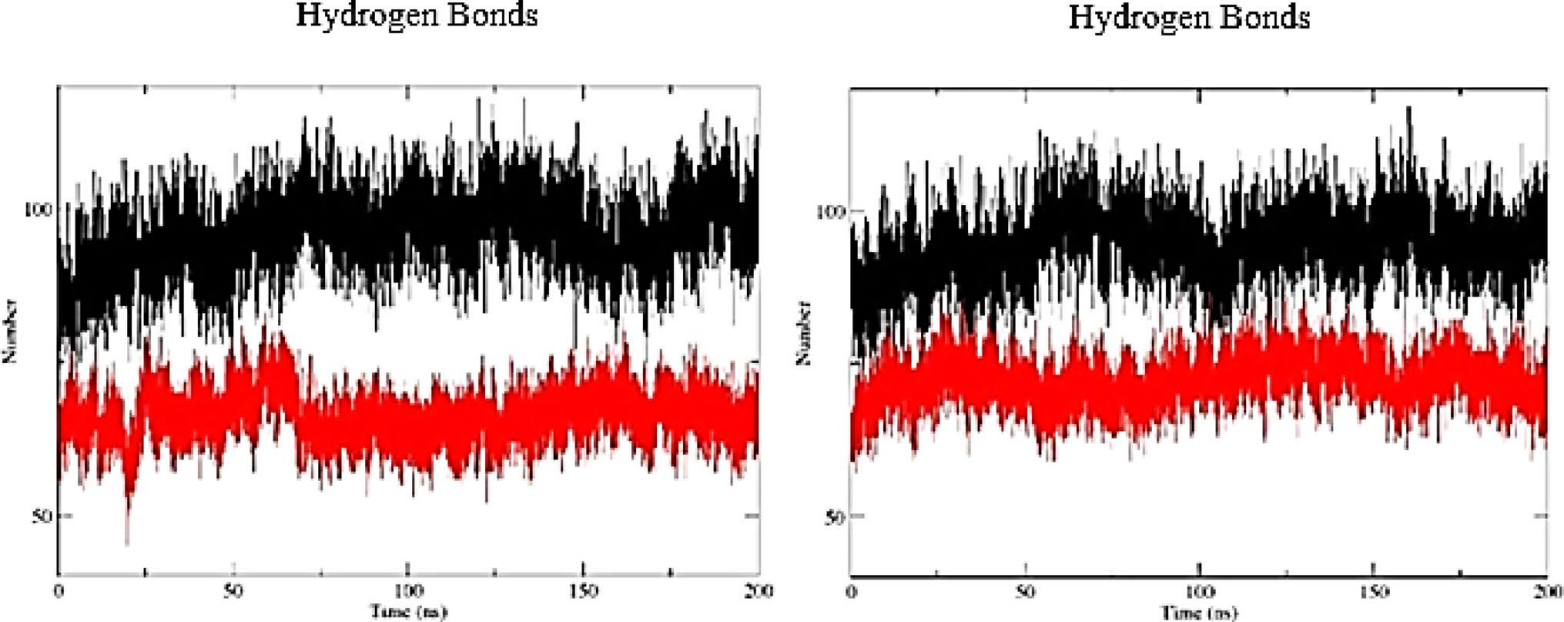
Time evolution of intramolecular hydrogen bonds for A11 and LN-A11 at **a** 300 K (26 °C) and **b** 310 K (36 °C). A11 is shown in black and LN-A11 in red

Similarly, analysis of intramolecular hydrogen bonds for the A11 aptamer revealed consistently higher average values compared with LN-A11 at both evaluated temperatures (Fig. 7; Table 1). This behavior suggests the maintenance of a more stable intramolecular interaction network for A11, whereas LN-A11 exhibits a lower density of hydrogen bonds and greater variability, indicative of increased structural mobility.

Although a slight increase in variability was noted when the temperature rose from 300 K to 310 K, the influence of temperature on hydrogen bond stability was minimal, indicating that the system remained relatively stable, with only minor losses or fluctuations at 310 K. This suggests that thermal resilience is likely maintained within this temperature range. Variations in hydrogen bonding patterns over time are expected in molecular dynamics simulations due to the intrinsic flexibility of biomolecular systems.

Notably, while this comparative analysis identifies A6 and A11 as the most stable molecules overall, both LN-A6 and LN-A11 exhibited well-defined plateaus during the simulations. This indicates that no significant fluctuations suggestive of structural instability were observed in the modified aptamers under the tested conditions.

Both RMSD and hydrogen bond analyses are essential metrics in molecular dynamics simulations to assess the conformational stability of biomolecules. Combining these approaches facilitates an understanding of the underlying structural and dynamic mechanisms, thereby contributing to advances in structural biology. In this study, a strictly computational comparison between ssRNA and ssDNA aptamers was conducted via molecular dynamics simulations, focusing on conformational preservation during ssRNA-to-ssDNA conversion.

Previous research has shown that converting ssRNA to ssDNA aptamers can confer higher chemical stability and bioavailability in biological environments [18]. However, our data provide additional evidence validating the applicability of this approach across different sequences derived from 3D Cell-SELEX, expanding the methodology’s scope. Complementarily, Jeddi and Saiz [2] corroborated the feasibility of integrating 3D structural modeling with molecular dynamics simulations to predict and maintain ssDNA aptamer conformational integrity, revealing that 3D structure preservation is crucial for maintaining functionality even after nucleic acid substitution.

## Conclusions

Structural modeling and molecular dynamics analyses enabled the characterization of the conformational properties of the A6 and A11 aptamers following conversion from ssRNA to ssDNA. Overall, ssRNA molecules exhibited greater structural stability than their ssDNA counterparts, as reflected by lower RMSD values and a higher number of intramolecular hydrogen bonds. However, these structural differences do not imply functional equivalence between the molecules.

Although molecular dynamics simulations do not allow direct inference of target-binding affinity, biological functionality, degradation rates, or economic feasibility – since detailed energetic analyses and experimental validations were not performed – the ssDNA aptamers exhibited consistent structural behavior at both evaluated temperatures (300 K and 310 K), with stable RMSD and hydrogen-bonding patterns, suggesting preservation of their three-dimensional architecture. Accordingly, the present study proposes a reproducible computational workflow to assess structural preservation during the conversion of aptamers from ssRNA to ssDNA, providing a methodological foundation for subsequent investigations. Future studies may incorporate energetic analyses, target-interaction simulations, and experimental validation to evaluate the functional relevance of these structures.

## Data Availability

No datasets were generated or analysed during the current study.
